# Femoral nerve block Intervention in Neck of Femur fracture (FINOF): study protocol for a randomized controlled trial

**DOI:** 10.1186/1745-6215-15-189

**Published:** 2014-05-24

**Authors:** Opinder Sahota, Martin Rowlands, Jim Bradley, Gerrie Van de Walt, Nigel Bedforth, Sarah Armstrong, Iain Moppett

**Affiliations:** 1 Department of Healthcare of Older People, Nottingham University Hospitals NHS Trust, Derby Road, Nottingham NG7 2UH, UK; 2 Anaesthesia and Critical Care Research Group, Division of Clinical Neuroscience, University of Nottingham, Queen’s Medical Centre, Derby Road, Nottingham, UK; 3 Queen’s Medical Centre campus, Nottingham University Hospitals NHS Trust, Derby Road, Nottingham NG7 2UH, UK; 4 NIHR Research Design Service for the East Midlands, Faculty of Medicine & Health Sciences, University of Nottingham, Queen’s Medical Centre, Derby Road, Nottingham NG7 2UH, UK

**Keywords:** Elderly, Hip fracture, Hip surgery, Analgesia, Femoral nerve block, Femoral nerve catheter, Nerve block

## Abstract

**Background:**

Hip fractures are very painful leading to lengthy hospital stays. Conventional methods of treating pain are limited. Non-steroidal anti-inflammatories are relatively contraindicated and opioids have significant side effects.Regional anaesthesia holds promise but results from these techniques are inconsistent. Trials to date have been inconclusive with regard to which blocks to use and for how long. Interpatient variability remains a problem.

**Methods/Design:**

This is a single centre study conducted at Queen’s Medical Centre, Nottingham; a large regional trauma centre in England. It is a pragmatic, parallel arm, randomized controlled trial. Sample size will be 150 participants (75 in each group). Randomization will be web-based, using computer generated concealed tables (service provided by Nottingham University Clinical Trials Unit). There is no blinding. Intervention will be a femoral nerve block (0.5 mls/kg 0.25% levo-bupivacaine) followed by ropivacaine (0.2% 5 ml/hr^−1^) infused via a femoral nerve catheter until 48 hours post-surgery. The control group will receive standard care. Participants will be aged over 70 years, cognitively intact (abbreviated mental score of seven or more), able to provide informed consent, and admitted directly through the Emergency Department from their place of residence. Primary outcomes will be cumulative ambulation score (from day 1 to 3 postoperatively) and cumulative dynamic pain scores (day 1 to 3 postoperatively). Secondary outcomes will be cumulative dynamic pain score preoperatively, cumulative side effects, cumulative calorific and protein intake, EUROQOL EQ-5D score, length of stay, and rehabilitation outcome (measured by mobility score).

**Discussion:**

Many studies have shown the effectiveness of regional blockade in neck of femur fractures, but the techniques used have varied. This study aims to identify whether early and continuous femoral nerve block can be effective in relieving pain and enhancing mobilization.Trial registration.

**Trial registration:**

The trial is registered with the European clinical trials database Eudract ref: 2010-023871-25. (17/02/2011). ISRCTN: ISRCTN92946117. Registered 26 October 2012.

## Background

Hip fractures remain one of the most serious injuries that occur in older people [1-3], with a mortality rate of 10% at one month, 20% at four months and 30% at one year [4]. Many of those who recover suffer a loss in mobility and independence. Approximately half of patients who were previously functionally independent become partly dependent, while one third become totally dependent [5]. The mean age of these patients is 81 years, 75% are female, and they are one the frailest groups of patients to be admitted to hospital, commonly with multiple co-morbidities. Hip fractures are painful [6], in both the pre and postoperative period. Adequate treatment of pain is not only a humanitarian issue, but may also impact on recovery. Pain is associated with increased neuro-hormonal stress response, myocardial ischemia, and delayed mobilization, all of which may increase postoperative length of stay and are associated with increased postoperative mortality. Untreated pain is also associated with delirium [7,8]. Current methods of providing analgesia include: paracetamol, non-steroidal anti-inflammatory drugs (NSAIDS), oral or parenteral opioids, and regional anaesthesia techniques. Paracetamol is an effective and safe analgesic [9], but insufficient for a significant number of patients when used alone. NSAIDs are largely contraindicated in this patient group due to their nephrotoxicity, and gastrointestinal side effects. They may also increase operative blood loss.

Opioids provide reasonable analgesia at rest but are relatively ineffective for dynamic pain (pain on movement). This is an issue postoperatively as patients often feel comfortable at rest but complain of moderate to severe pain during physiotherapy and ambulation. Opioid-related side effects are very common, distressing, and potentially serious. Nausea, vomiting, constipation, and delayed gastric emptying are common. Less common but more serious side effects include, delirium, respiratory depression, and death. Regional anaesthesia offers an attractive alternative to systemic opioids both for pre and postoperative use. Evidence from elective lower limb arthroplasty suggests that postoperative nerve block analgesia is beneficial overall [10] and may even enhance early mobilization, assuaging concern that impairment of motor function leads to a delay in ambulation [11]. However, to date, trials have been inconclusive regarding the benefit in acute hip fracture patients [12,13].

Several factors may explain this. Firstly, the historical success rate of regional anaesthetic techniques appears to have been relatively low. A non-randomized study of fascia iliaca block by Emergency Department physicians found adequate block at 1 hour in only 30% of subjects, although improvements in outcome (length of stay) were favorable in those with a successful block [12]. Use of ultrasound would be expected to improve the success rate of regional techniques and evidence does support this [14]. A recent trial comparing ultrasound-guided femoral nerve block to parenteral opioids in an Emergency Department setting in exactly this patient group demonstrated significantly reduced pain scores and decreased the need for rescue analgesia [15].

Another reason for the conflicting evidence is that most studies have provided a single injection nerve block either on admission or in the immediate perioperative period. Given that the pain experienced following hip fracture persists for several days, single injection nerve blocks may be inadequate as they only provide analgesia for up to 24 hours. There are two potential options to improve the duration of analgesia. Foss *et al.*[16] reported the successful use of low dose continuous epidural analgesia. Unfortunately this is not thought to be feasible or optimal in the United Kingdom at present for this group of patients. Another option is to provide initial analgesia with a single shot nerve block followed by continuous perineural infusion of local anaesthetic. One study which used postoperative perineural catheter infusion failed to show benefit [5], however, catheters were only inserted postoperatively. We therefore propose to study the effects of early and continuous femoral nerve block analgesia on dynamic pain and early rehabilitation compared to standard analgesic care.

## Methods/Design

### Primary aims

The primary aim of this study is to investigate if early use of femoral nerve blockade, with subsequent insertion of a femoral nerve catheter and an infusion of local anaesthetic, results in an increase in cumulative mobility score and a decrease in cumulative dynamic pain score in the first three postoperative days after surgery for a fractured neck of femur.

### Secondary aims

To investigate the early use of femoral nerve block results in a decrease in cumulative pain score (in the first 180 minutes of admission), a decrease in cumulative side effects (Nausea, vomiting, constipation and delirium) in the first three days post operatively, a decrease in overall length of stay, better calorific and protein intake and improved health related quality of life.

### Study design

This is a pragmatic, parallel arm, randomized controlled trial. The setting will be Queen’s Medical Centre Nottingham, a university teaching hospital and regional trauma centre in England.

### Randomization and blinding

There is no blinding and investigators and patients will be aware of their treatment group allocation. ‘Sham’ block and catheter insertion carries risks without analgesic benefit and as such was considered unethical. Randomization will take place in the Emergency Department as soon as patients have given verbal consent to take part in the trial. Randomization will be via password protected web-based randomization service provided by Nottingham University Clinical Trials Support Unit.

### Selection and withdrawal of patients

#### Recruitment

Patients presenting to the hospital with a suspected neck of femur fracture will be approached by a member of the Emergency Department staff, informed of the study, and referred to the research team for screening.

#### Inclusion criteria

Patients will be included if they are aged over seventy years old, previously resident in their own home, cognitively intact, previously independently mobile indoors and willing and able to give informed consent [17].

#### Exclusion criteria

Patients will be excluded if the were hospitalized at the time of fracture, have contraindications to femoral nerve blockade, are taking regular pre fracture opioids or glucocorticoids, have a history of alcohol or other substance abuse, have a documented serious adverse reaction to morphine, will have restrictions to their post operative mobilisation or are already participating in another clinical trial. Patients who, in the opinion of the investigator, have any condition which would adversely affect the study may also be excluded.

#### Informed consent

Patients will be assessed by the investigators regarding ability to give informed consent. An abbreviated mental test will be performed and patients scoring less than seven (out of ten) will be excluded. An acute hip fracture is extremely painful, therefore it is considered unethical to delay immediate treatment. Patient consent will be undertaken in two stages.

#### Stage 1

Subjects will be verbally informed of the exact nature of the study; the implications and constraints of the protocol; the known side effects; and any risks involved in taking part. It will be clearly stated that the participant is free to withdraw from the study at any time, for any reason, without prejudice to future care and with no obligation to give the reason for withdrawal. Verbal consent will be taken in the presence of at least one witness and recorded in writing in the medical notes.

#### Stage 2

At 48 hours following administration of the nerve block or standard analgesia, patients will be asked to give written informed consent to continue with the trial. The participant must personally sign and date the latest approved version of the informed consent form. The person obtaining consent will be a qualified and experienced member of the research team. A copy of the signed informed consent will be given to the participants and the original signed form retained within the study file. In an instance where the patient loses capacity to consent during the study, the researchers will ask for written informed consent from the next of kin (or if there is no next of kin the orthopedic consultant under whom the patient is currently treated). The proxy must personally sign and date the latest approved version of the proxy informed consent form.

#### Study intervention

Participants will be randomized into one of two arms: femoral nerve block followed by insertion of a femoral catheter and continuous femoral nerve blockade until 48 hours postoperatively; or standard analgesic care.

The femoral nerve block will be administered in the Emergency Department by the research anaesthetic fellow under ultrasound guidance (0.5 ml/kg^−1^ of 0.25% levo-bupivacaine up to 30 ml maximum volume). Immediately after radiographic confirmation of the fracture the participant will be transferred to the anaesthetic suite for insertion of femoral nerve catheter. Local anaesthetic will be infused using an elastomeric pump. This is a small plastic container housing an elastomeric bag containing the local anaesthetic. The elastic recoil of the bag drives the local anaesthetic through a resistor at a constant rate. The pump is small and light and does not require electrical power. The pump will contain 0.2% ropivacaine infusing at rate of 5 ml/^hr-1^; the dose of local anaesthetic to be used is based on local experience to provide analgesia without excessive motor block. Problems related to the pump or femoral catheter will be referred to the anaesthetic research fellow. In addition, subjects will be prescribed paracetamol (1 g) and tramadol (50 to 100 mg) every 6 hours. Breakthrough pain will be treated with oral morphine liquid (10 to 20 mg) every four hours.

#### Standard analgesic care

In the Emergency Department, participants will receive intravenous morphine titrated to a visual analogue score of five or less at rest. Regular paracetamol (1 g every 6 hours) and tramadol (50 to 100 mg every 6 hours) will be prescribed. Breakthrough pain will be treated with oral morphine liquid (10 to 20 mg every four hours) as required. The dose and duration of the tramadol will be reviewed daily postoperatively and decreased according to patient tolerance.

#### Concomitant medication

Patients who are on regular pre-fracture opioid therapy will be excluded. Pre-fracture medication will be reviewed by the orthogeriatrician and continued or amended as clinically appropriate for the individual patient. Medication started while in hospital will be decided by the attending orthogeriatrician as required by the patient’s condition. Non-steroidal anti-inflammatory drugs are only used in exceptional circumstances in this group of patients at our institution.

#### Concomitant treatments

The type of anaesthesia will be at the discretion of the attending anaesthetist on the day of surgery. Common practice at our institution is to place a femoral nerve block either to facilitate patient positioning for a spinal anaesthetic or as postoperative analgesia in patients having general anaesthesia. This will be permitted in patients in the control group, however catheter insertion is not. If the patient is in the intervention group the block may be augmented using the catheter prior to surgery.

#### Standard care

Standard care will otherwise be identical in both groups in accordance with national guidelines and standards [18,19]. All patients are admitted to dedicated trauma wards and cared for under a hip fracture pathway. This includes rapid assessment and admission from the Emergency Department, intravenous fluids from the time of admission, assessment by orthogeriatricians, operation within 36 hours of admission, assessment of bone health and falls, multi-professional care, and discharge planning. Operations are performed in dedicated trauma theatres by consultants or senior trainees in anaesthesia and orthopedic trauma.

#### Statistics

Data will be collected and inputted into an electronic database by the research team. Analysis will be performed by the trial statistician, using the latest version of IBM© SPSS© Statistics software (Copyright IBM Corporation 2012). There will be no interim analysis.

#### Sample size and justification

Based on mean cumulative postoperative mobility (CAS) score, and mean cumulative postoperative dynamic pain score from the study by Foss *et al.*[16], and standard deviations estimated by the formula ‘range/6’, for the CAS outcome measure, a sample size of 37 participants per group would be required to detect a 2 point difference in mean scores between the nerve block and standard analgesic group (2 point difference shown to be associated with a change in postoperative length of stay), assuming a two-sided significance level of 0.05 and 80% power. For the cumulated pain score, a sample size of 67 participants per group would be required to detect a 2.5 point difference in mean scores between the two groups, using a 10 point pain scale (1 point difference shown to be statistically significant in the study by Foss *et al.*[16], using a 4 point pain scale).

Recruiting 75 patients per arm, allows a detection of a 1.5 point difference in the CAS score and 2.5 point difference in the cumulative pain score, with a 10% attrition rate, assuming a two-sided significance level of 0.05 and 80% power.

### Definition of datasets analyzed

The safety set will comprise all randomized participants who receive femoral nerve block or are randomized to standard treatment. The full analysis set will comprise all randomized participants. The per-protocol set will comprise all participants in the full analysis set who are deemed to have no major protocol violations that could interfere with the objectives of the study.

#### Reporting of adverse events

Patients will be seen daily while in the study. All adverse events will be recorded and closely monitored until resolution or stabilization, or until it has been shown that the study intervention is not the cause. The chief investigator will be informed immediately of any serious adverse events and shall determine the seriousness and causality in conjunction with any treating medical practitioners. All treatment-related serious adverse events will be recorded and reported to the research ethics committee as part of annual reporting. Unexpected serious adverse events will be reported to the ethics committee and sponsor within the relevant time frames. The chief investigator will be responsible for all adverse event reporting.

### Ethics committee and regulatory approval

The trial received ethical approval from Nottingham Research Ethics Committee on 28 January 2011. (Reference 10/H0408/113). The trial is funded by NIHR Project Number: PB-PG-0909-19114. The trial will be conducted in accordance with the Declaration of Helsinki 1996, principles of good clinical practice, and the Department of Health Research Governance Framework for Health and Social Care.

## Discussion

There seems little doubt that regional analgesia is effective [11,15,16], however, uncertainty remains surrounding the optimal regional anaesthetic technique and its timing.

The popularity and development of ultrasound guidance in regional anaesthesia has demonstrated that block effectiveness can be increased [14] and that these blocks can be placed relatively quickly. Many studies, including the one by Haines *et al.*[14], placed their blocks after radiological confirmation of fracture. While this may seem a sensible approach many patients do experience severe pain during movement of the fractured limb in the X-ray department. This was reinforced in the 2011 UK NICE guidelines:

‘It must be remembered that patients may require more analgesia for investigations such as X-Rays.’ [19]

This is why, for our study, randomization and initial block occurs before the patient has any radiological intervention. This does, however, raise different issues. Firstly, inserting a nerve block for a patient whose X-rays subsequently reveal no hip fracture. This has the potential to precipitate an admission to hospital. Secondly, it is possible to miss patients who present atypically, and are later revealed to have a hip fracture.

Both these issues would need addressing if this intervention were to be implemented as standard practice. Regarding the first scenario, for trial purposes, patients who have no hip fracture are excluded from further analysis. We are aware of a few patients who have been found not to have an overt hip fracture. Some have fractures of the pubic ramus or acetabulum instead, and are not suitable for discharge as their mobility is significantly compromised and they are in significant pain. A small number have required admission for further investigation of subtle radiographic abnormalities. In this situation block insertion has not delayed discharge.

Due to the requirement to place the initial block quickly we have divided our consent into two stages. Witnessed verbal consent prior to initial block placement, followed by written consent after 48 hours. This has occasionally resulted in patients giving verbal consent initially but then becoming drowsy or confused postoperatively and being unable to sign a formal written consent. In these instances we have retained the patients in their allocation on an intention-to-treat basis and obtained proxy consent from a relative. While full formal consent from the beginning should be the gold standard we felt that delaying initial analgesia would be unethical. Also we believe that development of delirium pragmatically reflects the behaviour of this cohort of patients. Not providing analgesia can also have similar consequences [7].

Dislodgement of catheters is a problem with continuous nerve blockade. For our study we took the pragmatic approach that if the catheter became dislodged within 24 hours of operation then it would be re-sited, but beyond this the patient would be moved to standard care. They would remain in the intervention group on an intention-to-treat basis. We believe that this reflects realistic practice at our unit and others.

## Trial status

The study is currently ongoing. The first patient was randomized on 6 January 2012. A total of 120 participants have been recruited so far, with an estimated completion date of June 2014.

## Abbreviations

CAS: Cumulative ambulation score; NICE: National institute of clinical excellence; NSAIDS: Non steroidal anti-inflammatory drugs.

## Competing interests

The authors declare that they have no competing interests.

## Authors’ contributions

OS designed and initiated the study and reviewed the manuscript. IM designed and initiated the study and reviewed the manuscript. NB designed and initiated the study and reviewed the manuscript. MR performed the blocks and catheters and drafted the manuscript. JB performed the blocks and catheters. GVdeW performed the blocks and catheters. SA provided statistical knowledge for study initiation and will perform the statistical analysis. All authors read and reviewed the final manuscript.
